# The Pulmonary Metastasectomy in Colorectal Cancer cohort study: Analysis of case selection, risk factors and survival in a prospective observational study of 512 patients

**DOI:** 10.1111/codi.15651

**Published:** 2021-05-05

**Authors:** Tom Treasure, Vernon Farewell, Fergus Macbeth, Tim Batchelor, Mišel Milošević, Juliet King, Yan Zheng, Pauline Leonard, Norman R. Williams, Chris Brew‐Graves, Lesley Fallowfield, the PulMiCCinvestigators

**Affiliations:** ^1^ Clinical Operational Research Unit University College London London UK; ^2^ MRC Biostatistics Unit Cambridge UK; ^3^ Centre for Trials Research Cardiff University Cardiff UK; ^4^ Bristol Royal Infirmary University Hospitals Bristol UK; ^5^ Institute for Lung Diseases of Vojvodina Thoracic Surgery Clinic Sremska Kamenica Serbia; ^6^ Thoracic Surgery Guy's and St Thomas' Hospital London UK; ^7^ Department of Thoracic Surgery Affiliated Cancer Hospital of ZhengZhou University/Henan Cancer Hospital Zheng Zhou China; ^8^ Barking, Havering and Redbridge University Hospitals Romford UK; ^9^ Surgical and Interventional Trials Unit (SITU) University College London London UK; ^10^ National Cancer Imaging Accelerator (NCIA) Division of Medicine University College London London UK; ^11^ Sussex Health Outcomes Research and Education in Cancer (SHORE‐C) University of Sussex Falmer UK

**Keywords:** lung metastasectomy, prospective observational study

## Abstract

**Aim:**

We wanted to examine survival in patients with resected colorectal cancer (CRC) whose lung metastases are or are not resected.

**Methods:**

Teams participating in the study of Pulmonary Metastasectomy in Colorectal Cancer (PulMiCC) identified potential candidates for lung metastasectomy and invited their consent to join Stage 1. Baseline data related to CRC and fitness for surgery were collected. Eligible patients were invited to consent for randomization in the PulMiCC randomized controlled trial (Stage 2). Sites were provided with case report forms for non‐randomized patients to record adverse events and death at any time. They were all reviewed at 1 year. Baseline and survival data were analysed for the full cohort.

**Results:**

Twenty‐five clinical sites recruited 512 patients from October 2010 to January 2017. Data collection closed in October 2020. Before analysis, 28 patients with non‐CRC lung lesions were excluded and three had withdrawn consent leaving 481. The date of death was known for 292 patients, 136 were alive in 2020 and 53 at earlier time points. Baseline factors and 5‐year survival were analysed in three strata: 128 non‐randomized patients did not have metastasectomy; 263 had elective metastasectomy; 90 were from the randomized trial. The proportions of solitary metastases for electively operated and non‐operated patients were 69% and 35%. Their respective 5‐year survivals were 47% and 22%.

**Conclusion:**

Survival without metastasectomy was greater than widely presumed. Difference in survival appeared to be largely related to selection. No inference can be drawn about the effect of metastasectomy on survival in this observational study.

**What does this paper add to the literature?**

The assumed near‐zero survival without resection of patients with lung metastases from colorectal cancer was not supported by this study. It seems likely that a much smaller part of the 40% observed 5‐year survival can be attributed to lung metastasectomy than is widely believed.

## INTRODUCTION

The Pulmonary Metastasectomy in Colorectal Cancer (PulMiCC) study enrolled 512 patients who were being considered for lung metastasectomy from October 2010 to January 2017. Nested within this observational study was the PulMiCC randomized controlled trial (RCT) of 93 patients. The RCT was reported in *Colorectal Disease* in 2020 [1]. The widely believed large survival benefit from lung metastasectomy was not evident although a small late effect cannot be excluded. Patient‐reported outcomes—quality of life and health utility—declined at a similar rate in the metastasectomy and the unoperated control arms [2, 3].

**FIGURE 1:**
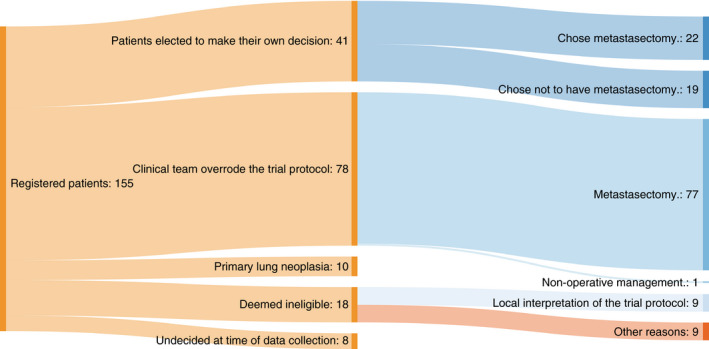
Concerned by the low rate of randomization into the RCT the Independent Data Monitoring Committee asked for an investigation. The three largest recruiting centres (Bristol, Liverpool and Sheffield) provided reasons for not randomizing in 155 patients. Among 78 patients for whom the MDT overrode the patients' wishes to be in a controlled trial, 77 (99%) were operated on. When patients made their own decision they were more evenly divided, demonstrating group equipoise. The results are given in the Sankey chart. Diagram created using SankeyMATIC (http://sankeymatic.com/)

The PulMiCC study was run in the context of a firm belief in the clinical effectiveness of lung metastasectomy. In response to a paper arguing the case for a trial [4], the *European Journal of Cardio‐Thoracic Surgery* published an Editorial in 2017 proclaiming ‘Surgery for pulmonary metastases is a pillar of modern thoracic surgery’ [5]. In support of lung metastasectomy for colorectal cancer (CRC), the authors cited two retrospective single‐institution studies [6, 7]. These provided a pooled 5‐year survival of 60% in groups of 165 and 113 patients collected over 10 and 12 years. The Work Force of Evidence Based Surgery of the Society of Thoracic Surgeons based its recommendations for lung metastasectomy on the assumption that 5‐year survival without resection is zero [8]. Neither statement was supported by control data. These publications in the leading thoracic surgical journals invite the conclusion that lung metastasectomy provides 60% survival benefit. This widely held belief and the climate of certainty resulted in multidisciplinary teams (MDTs) having difficulty randomizing patients into the PulMiCC trial (Figure 1).

Although PulMiCC found no difference in survival, a small survival advantage due to resection of lung metastases that prove to be the only site of residual CRC cannot be discounted. But the trial was large enough to refute the improbable 0% [8] and the less extreme estimate of 5% more generally cited [9]. The 5‐year survival in the control group was 30% (95% CI 15.3–45.7%), significantly above 5% (*P* < 0.001) [1].

*Colorectal Disease* has recently published a big data study of pulmonary metastasectomy for CRC [10]. Among 173 354 patients who had a CRC resection from 2005 to 2013, lung resection was subsequently reported in 3434 patients which averages 2% over the time span of the study. From this an average number of pulmonary metastasectomy operations for CRC can be estimated at about 380 per annum in the National Health Service (NHS) in England. During an overlapping period from late 2010 to early 2017, the PulMiCC cohort recruited more than 300 patients who had a lung metastasectomy for CRC which is equivalent to about 14% of all patients having this procedure in England.

Here we report data from the full PulMiCC cohort. The large majority of the patients were not randomized and so we cannot draw any valid inferences about survival attributable to lung metastasectomy. Nevertheless, the characteristics of the large sample of patients and their outcomes are available to inform the Association of Coloproctology of Great Britain and Ireland (ACPGBI) IMPACT initiative and the design of future research in this uncertain area [11, 12].

## GENERAL METHODS

### Recruitment

The PulMiCC cohort study was planned around the PulMiCC RCT. The study was administered by clinical trials staff based at the recruiting hospitals under the direction of the site's principal investigator (PI) who was either a thoracic surgeon or an oncologist. Patients who were potential candidates for lung metastasectomy were given written information and an explanatory DVD to take home. A healthcare professional training DVD was also available for clinicians to aid their discussions with patients.

Interested patients were invited to sign Stage 1 to be assessed for lung metastasectomy within PulMiCC and to be registered by the trials unit. They consented to collection of baseline information: sex, age, height and weight to derive body mass index, the interval since primary CRC resection, whether they had prior liver metastasectomy, the number of lung metastases, carcinoembryonic antigen assay (CEA) and tests of lung function.

Stage 1 consent included 1‐year follow‐up by a Case Report Form (CRF) to record treatments since evaluation, including lung metastasectomy and other cancer directed local interventions. The sites were given CRFs to report death, serious adverse events, new lung metastases, new liver metastases and other cancer recurrence.

The local MDT considered whether patients should have lung metastasectomy and, if they were uncertain, agreed to offer patients randomization in the PulMiCC RCT. When the PulMiCC was closed, in view of the size and wealth of baseline data about the 82.2% non‐randomized patients we sought Research Ethics Committee agreement to approach sites for follow‐up data. Approval was readily granted as an audit of practice on 11 February 2019. All patients who gave consent on entry to Stage 1 are accounted for in this analysis.

From 11 February 2019, all the site PIs and trials staff were approached with an individualized CRF request for each patient in Stage 1 asking for a date of death or when last known alive. We repeated the requests for missing survival information until the end of October 2020.

### Statistical methods

Data from the full PulMiCC cohort were used to investigate baseline factors that influenced survival. Patients not known to have died were censored at the last time they were known to be alive in all analyses of survival. These investigations were based on Cox's relative risk model with time to death as the outcome and time from cohort entry as the time scale. The observational nature of the cohort does not support the estimation of treatment effects. Three strata were defined and all analyses were stratified on this basis.

1. The first stratum included all patients who were not randomized and did not have a metastasectomy. They entered the analysis from their time of cohort entry.
2. Patients receiving an elective metastasectomy formed the second stratum, entered at the time of their operation.
3. Patients in the RCT formed the third stratum entering at the time of randomization.

Results are summarized as hazard ratios (HRs) with confidence intervals (CIs). An HR greater than 1 indicates that larger values of the risk factor are associated with poorer survival and an HR less than 1 indicates that larger values are associated with higher survival. For categorical risk factors, an HR >1 indicates that poorer survival is associated with the presence of the risk factor (e.g., for male sex) or with a risk factor category compared to the reference category as for performance status using the Eastern Cooperative Oncology Group (ECOG) scores.

For descriptive purposes, survival curves were also estimated based on the fixed grouping of the patients into these three strata. The times of origin for these three curves are different and are the dates of cohort entry. These classifications are retrospective and therefore analyses using these from the time of cohort entry are ad hoc because the classes are determined after cohort entry and, more importantly, by decisions made by the patient and MDT following cohort entry.

## RESULTS

### Sources of patients and composition of the three strata

In all, 512 patients were recruited into Stage 1 of the PulMiCC trial from October 2010 to January 2017 from 25 clinical sites, mainly in England but also in China, Scotland, Serbia and Sicily (Table 1). Of the 512 patients, 31 were excluded for reasons given in Table 2. This left 481 patients in the cohort, of whom 90 had been randomized in the PulMiCC RCT. Of these, 41 never had a metastasectomy and 49 had a lung metastasectomy 0 to 627 days after randomization (median 51; interquartile range 27–160 days) including some late crossovers.

**TABLE 1:** Trials sites and numbers of participants included at each site

Site	City and institution	Participants enrolled
076	Sheffield, Northern General Hospital	101
073	Liverpool, Heart and Chest Hospital	88
062	Bristol, Royal Infirmary Serbia, Institute for Lung Diseases of Vojvodina Middlesbrough, James Cook Hospital Cambridge, Papworth Hospital	77
078		41
070		29
029		29
028	London Guy's Hospital	22
060	Basildon, Basildon Hospital	15
065	Plymouth, Derriford Hospital	15
068	Glasgow, Golden Jubilee Hospital	15
071	Sutton in Ashfield, King's Mill Hospital	12
063	Manchester, Christie	11
067	Leicester, Glenfield Hospital	10
074	Wolverhampton, New Cross Hospital	9
105	Zhengzhou, Henan Cancer Hospital	6
066	Newcastle, Royal Victoria Hospital	5
075	Norwich, Norfolk and Norwich Hospital	5
080	Catania, Policlinico Hospital	5
082	London, Royal Brompton Hospital London, Royal Free Hospital Belfast, City Hospital	5
018		4
061		2
069	Birmingham, Heartlands Hospital	2
084	London, St George's Hospital	2
072	Leeds, St James' Hospital	1
081	Burton, New Cross Hospital	1
	Total patients	512

**TABLE 2:** Reasons for exclusion

Total Stage 1 Recruitment		512
Reasons for exclusion		
Non‐neoplastic nodules	13	
Primary lung cancer	13	
Carcinoid tumour	1	
Hamartoma	1	
Consent withdrawn	3	
Total exclusions		31
Included patients		481

**FIGURE 2:**
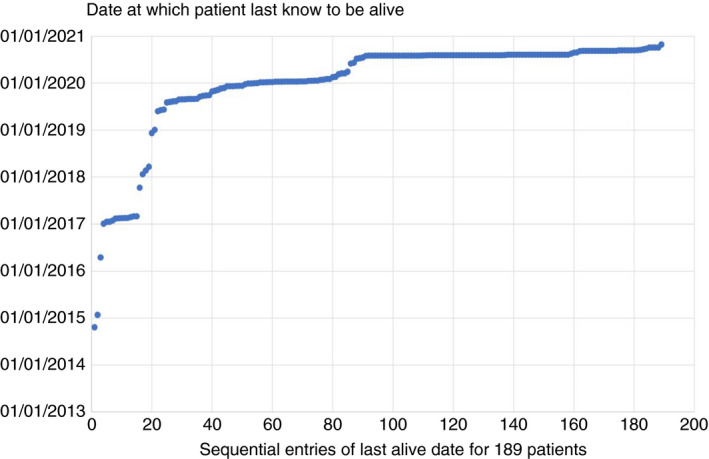
For 189 patients where a death form had not been returned this chart gives the date at which they were last known to be alive in order from the earliest to the most recent. Requests continued until October 2020 when we closed the study for analysis. Some centres regarded the close of the RCT as the end of their commitment to give time to the PulMiCC study

**FIGURE 3:**
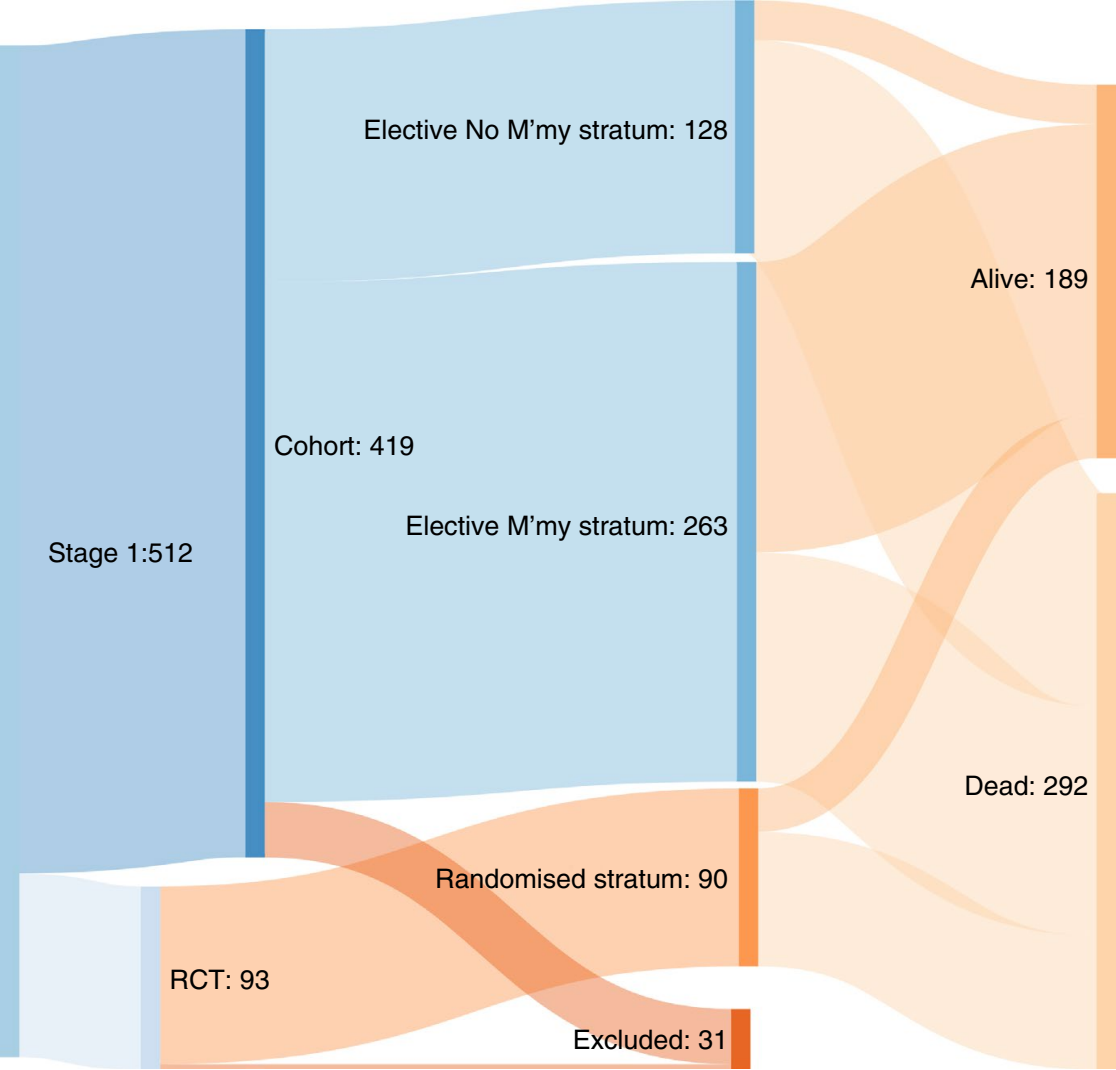
The total 512 Stage 1 enrolled patients divide into 93 in the RCT and the remaining 419 in the observational study. After 31 exclusions for the reasons given in Table 2, 481 patients from the PulMiCC cohort are analysed in three strata: elective non‐metastasectomy 128, elective metastasectomy 263 and the 90 patients who were randomized. Diagram created using SankeyMATIC (http://sankeymatic.com/)

We received a record of the date of death for 292 of them. Four patients who had metastasectomy died on the day of surgery. For the 189 patients without death dates, 136 were known to be alive in 2020, 33 in 2019 and for 20 patients last alive dates were longer ago than the end of December 2018 (Figure 2, Table 3). Data collected up to October 2020 are used in this analysis. The source, mix and eventual outcome of the three strata are shown in Figure 3.

**TABLE 3:** Recorded date of alive or dead status in all 481 cohort patients

Status	From	To	Number
Known dates of death	31 July 2012	11 August 2020	292
Known to be alive to 2020	01 January 2020	29 October 2020	136
Known to be alive to 2019	03 January 2019	30 December 2019	33
Last known to be alive <2019	22 October 2014	10 December 2018	20
Total			481

### Mortality risk factors

**FIGURE 4:**
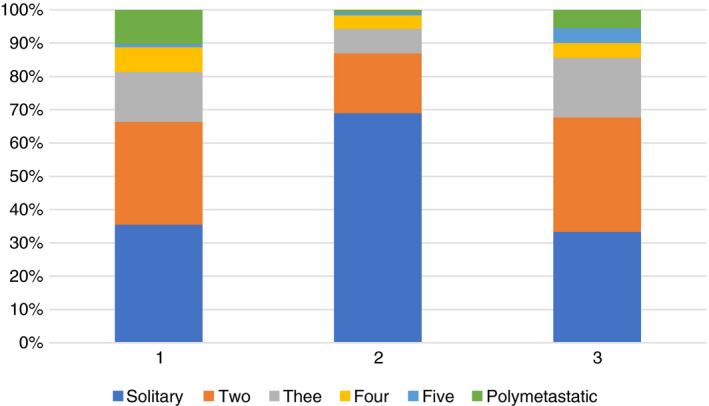
Three numbers of lung metastases in the three strata based on available data as in Table 5: (1) *N* = 128 non‐randomized patients who did not have a metastasectomy; (2) *N* = 263 non‐randomized patients who had a metastasectomy; (3) *N* = 90 randomized patients

The baseline risk factors for the patients in the three strata are shown in Table 4. The proportions of metastases in the three strata (Figure 4) show the predominance of solitary metastases (69%) in the elective metastasectomy group (Table 5).

**TABLE 4:** Data availability and median, full range and quartile values in the three strata

	Data available^a^	Percentage	Minimum	0.25	Median	0.75	Maximum
Age in years							
Elective no metastasectomy	128	99.2	42.4	65.7	71.9	77.7	87.9
Elective metastasectomy	263	100.0	30.8	60.0	67.0	72.8	85.6
Randomized	90	100.0	35.3	59.8	67.1	73.8	86.5
Body mass index							
Elective no metastasectomy	105	81.4	17.9	25.0	27.8	30.9	57.8
Elective metastasectomy	259	98.5	17.0	24.6	27.7	31.4	56.8
Randomized	86	95.6	18.7	26.1	28.6	31.7	40.9
Carcinoembryonic antigen, ng/l							
Elective no metastasectomy	82	63.6	0.3	1.7	3.0	5.9	57.0
Elective metastasectomy	144	54.8	0.3	1.3	2.0	4.0	151.0
Randomized	78	86.7	0.3	1.9	3.0	4.6	74.0
Forced expiratory volume, % predicted							
Elective no metastasectomy	90	70.3	44.0	76.0	86.6	101.5	145.0
Elective metastasectomy	233	88.6	26.0	82.3	96.0	111.0	153.0
Randomized	84	93.3	46.0	78.0	93.1	109.3	139.0
Months from primary CRC resection to metastasectomy or registration							
Elective no metastasectomy	117	91.4	1.3	13.9	25.6	49.7	145.4
Elective metastasectomy	253	96.2	0.0	14.3	24.7	37.1	138.7
Randomized	84	93.3	0.8	13.8	25.6	36.9	130.3
Number of metastases							
Elective no metastasectomy	107	82.9	1	1	2	3	17
Elective metastasectomy	245	93.2	1	1	1	2	8
Randomized	90	100.0	1	1	2	3	9
% solitary			Missing	Solitary	Multiple		% solitary
Elective no metastasectomy	107	83.6	22	38	69		35.5%
Elective metastasectomy	245	93.2	18	169	76		69.0%
Randomized	90	100.0	0	30	60		33.3%
ECOG			Missing	0	1	2	
Elective no metastasectomy	90	70.3	29.7%	35.9%	29.7%	4.7%	
Elective metastasectomy	215	81.7	18.3%	67.9%	31.6%	0.5%	
Randomized	74	82.2	21.6%	73.0%	25.7%	1.4%	
Sex			Women	Men		% women	
Elective no metastasectomy	128	100.0	42	87		36.4%	
Elective metastasectomy	263	100.0	100	163		38.0%	
Randomized	90	100.0	34	56		37.8%	
Prior liver metastasectomy			Yes	No		% previous	
Elective no metastasectomy	121	94.5	44	77		36.4%	
Elective metastasectomy	256	97.3	71	185		27.7%	
Randomized	83	92.2	26	57		31.3%	

Abbreviations: CEA, carcinoembryonic antigen; CRC, colorectal cancer; ECOG, Eastern Cooperative Oncology Group.

^a^ There are usually more missing data in the elective non‐metastasectomy group because, if metastasectomy is decided against for any reason, other investigations may serve no purpose. CEA is an exception. It was required for minimization but the value was not always in the record. It is a paradoxical marker because its elevation on screening may prompt a search for metastases but its elevation is a negative predictor for survival after metastasectomy and may contribute to a decision against operating.

**TABLE 5:** Numbers of patients with a solitary metastasis and 2, 3, 4, 5 and polymetastatic disease in each stratum

	Solitary	Two	Three	Four	Five	Polymetastatic	Available
Elective no metastasectomy	38	33	16	8	1	11	107/128
Elective metastasectomy	169	44	18	10	2	2	245/263
Randomized	30	31	16	4	4	5	90/90

Table 6 presents the results from single‐factor analyses of baseline factors derived from stratified Cox regression models. The table includes the number of deaths examined in each analysis as these vary depending on the number of patients for whom information is available on the various potential risk factors. The factors demonstrating evidence of a higher mortality risk were higher values of log(CEA), a shorter interval from CRC to cohort entry and higher ECOG classes with a suggestive effect for male sex. Multiple lung metastases and prior liver metastases also demonstrated limited potential for an increase in mortality so were also considered for multi‐factor analyses. There was some evidence that the effect for log(CEA) was different in different strata. Estimated HRs and 95% CIs were 1.73 (1.37, 2.19), 1.16 (0.93, 1.44) and 1.58 (1.22, 2.05) in the elective no metastasectomy, elective metastasectomy and randomized groups respectively with no effect being demonstrable in the elective metastasectomy group.

**TABLE 6:** Results from single‐factor analyses using a stratified Cox regression model

Variable	Categories	No. of deaths	HR (95% CI)	*P* value
Age (continuous)		292	1.00 (0.99,1.01)	0.73
Male sex		292	1.24 (0.98,1.58)	0.08
Log(CEA)		200	1.42 (1.25,1.62)^a^	<0.001
Liver metastasectomy (Y/N)		280	1.22 (0.95,1.56)	0.12
No. of lung metastasectomies > 1		265	1.19 (0.92,1.54)	0.181
Interval CRC to start (years)		274	0.90 (0.84,0.97)	0.003
BMI		269	1.00 (0.98,1.02)	0.99
ECOG	0	219	1.00	
	1		1.28 (0.97,1.69)	0.09
	2		1.11 (0.49,2.56)	0.80
%FEV		247	1.00 (0.99,1.00)	0.20

Note

Strata: elective no metastasectomy or randomization; elective metastasectomy; randomized.

Abbreviations: BMI, body mass index; CEA, carcinoembryonic antigen; CRC, colorectal cancer; ECOG, Eastern Cooperative Oncology Group; FEV, forced expiratory volume in the 1st second; HR, hazard ratio.

^a^ Some evidence of effects being different in different strata.

Table 7 presents results from multi‐factor stratified Cox regression models including variables demonstrating some potential for a relationship in single‐factor analyses. The set of models presented is based on gradually decreasing numbers of deaths depending on the availability of risk factor information for the variables included with a final model with the four most significant variables. These were male sex, interval (shorter) from CRC to cohort entry, prior liver metastases and log(CEA). Again, there was evidence of differential effects for log(CEA) with estimated HRs and 95% CIs of 1.57 (1.20, 2.04), 1.14 (0.92, 1.43) and 1.52 (1.16, 1.98) in the elective no metastasectomy, elective metastasectomy and randomized groups respectively.

**TABLE 7:** Results from multi‐factor analyses using a stratified Cox regression model

	Model A	Model B	Model C	Model D	Model E
No. deaths	274	270	251	191	192
Male sex	1.30 (1.02, 1.67) (*P* = 0.036)	1.29 (1.00, 1.65) (*P* = 0.051)	1.20 (0.93, 1.55) (*P* = 0.167)	1.25 (0.93, 1.69) (*P* = 0.144)	1.45 (1.07, 1.98) (*P* = 0.018)
Interval CRC to start	0.90 (0.84, 0.96) (*P* = 0.002)	0.88 (0.81, 0.94) (*P* < 0.001)	0.89 (0.82, 0.95) (*P* < 0.001)	0.86 (0.79, 0.94) (*P* < 0.001)	0.84 (0.77, 0.92) (*P* < 0.001)
Liver metastasectomy (Y/N)		1.33 (1.02, 1.74) (*P* = 0.035)	1.27 (0.96, 1.67) (*P* = 0.092)	1.23 (0.89, 1.70) (*P* = 0.210)	1.29 (0.94, 1.76) (*P* = 0.113)
No. of lung metastasectomies >1			1.19 (0.91, 1.55) (*P* = 0.208)	1.23 (0.90, 1.68) (*P* = 0.191)	
ECOG (0)				1.00	
ECOG (1)				1.31 (0.97, 1.78) (*P* = 0.081)	
ECOG (2)				0.96 (0.38, 2.44) (*P* = 0.938)	
Log(CEA)					1.35 (1.17, 1.55) (*P* < 0.001)

Note

Strata: elective no metastasectomy or randomization; elective metastasectomy; randomized. HR (95% CI) (*P* value).

Abbreviations: CEA, carcinoembryonic antigen; CRC, colorectal cancer; ECOG, Eastern Cooperative Oncology Group; HR, hazard ratio.

### Survival

**FIGURE 5:**
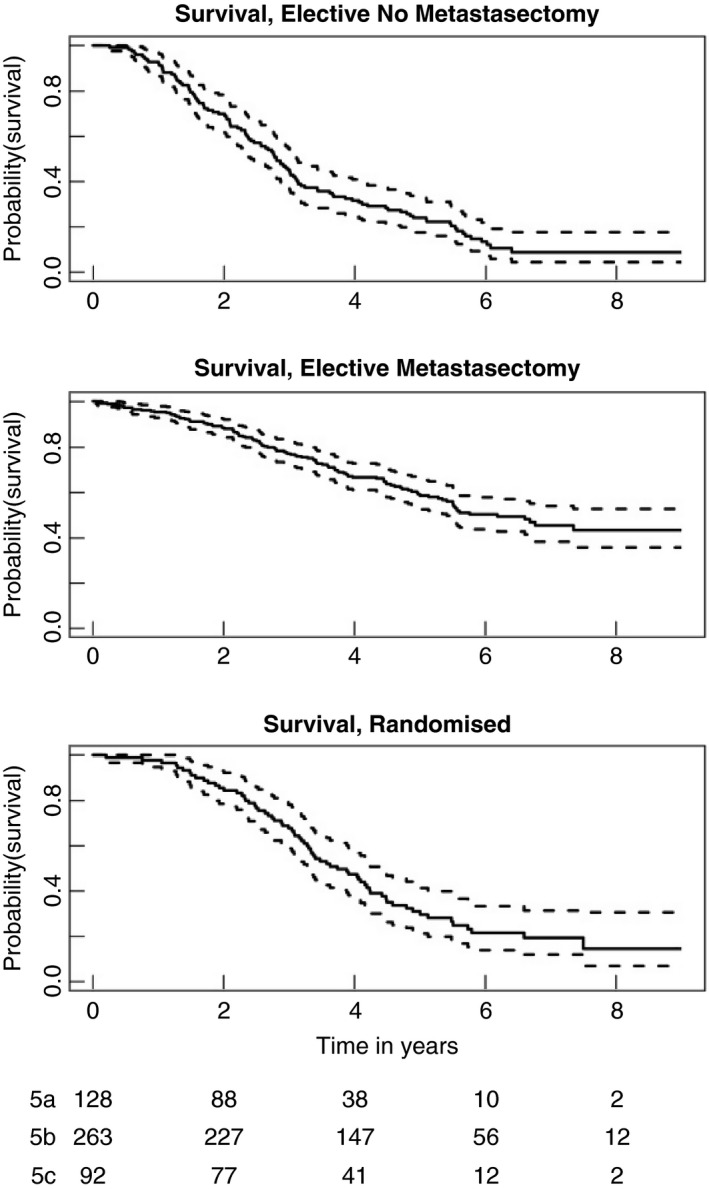
Survival in three strata with 95% confidence intervals. The curves are displayed separately because the entry points into the analyses and the baseline prognostic factors of those in the three strata are different. No direct comparison of these curves is appropriate

With a time origin of cohort entry, Figure 5 displays Kaplan–Meier estimated survival curves and 95% CIs of three groups of patients retrospectively classified in the strata: non‐randomized and no metastasectomy, elective metastasectomy, and randomized.

**FIGURE 6:**
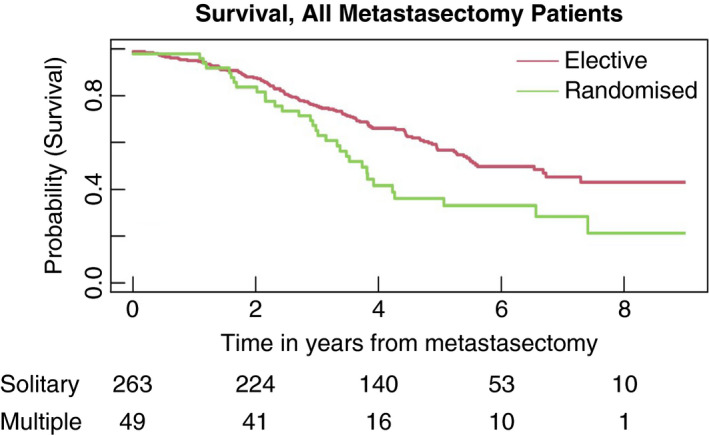
Survival curves for all 312 patients who had pulmonary metastasectomy separated into those in the elective and randomized strata

Figure 6 displays the estimated survival after metastasectomy curves for randomized and elective metastasectomy patients. The curve in Figure 6 begins at a value of 0.989 for elective patients because there were three patients with a death date on the same day as the day of metastasectomy. The curve for randomized patients begins at 0.980 with one death on the day of metastasectomy. Overall, in the 6 months after metastasectomy there were 9/288 deaths compared with 2/166 among those who did not have an operation in the 6 months after cohort entry.

**FIGURE 7:**
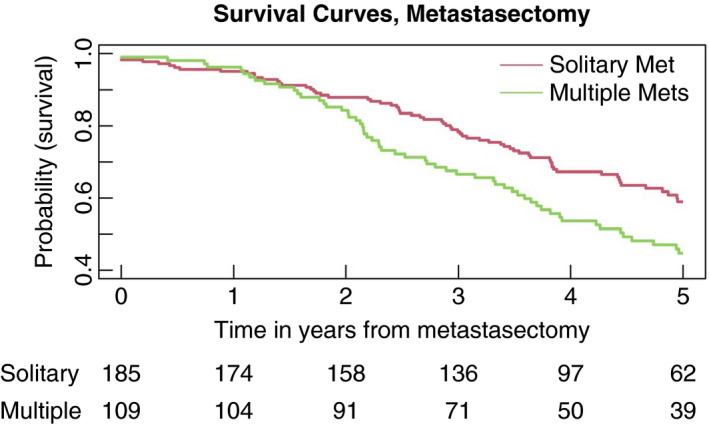
Survival for elective metastasectomy patients with solitary or multiple metastases. The difference in survival does not appear until nearly 2 years

Over the time period after metastasectomy, the overall estimated HR (95% CI) for multiple metastases vs. a single metastasis, stratified by elective/randomized status, in PulMICC patients having a metastasectomy is 1.33 (0.94, 1.88) (*P* = 0.10). The separate estimates based on elective and randomized metastasectomies are 1.18 (0.80, 1.76) (*P* = 0.40) and 2.15 (0.93, 4.99) (*P* = 0.07) respectively providing suggestive evidence for a much more marked effect in the randomized patients. The difference in survival for those having elective resection of solitary and multiple metastases is shown in Figure 7. The number of lung metastases is a risk factor of particular relevance for metastasectomy patients and is considered further in the next section.

### Interpretation of results

All the curves in Figure 5 show a relatively slow rate of decline initially. This is a hallmark of many cancer survival studies—whether randomized or observational—in contrast to cancer registry data presentations when there is characteristically an early sharp fall which becomes progressively flatter. For randomized patients, the plateau may be because patients have to have a good probability of short‐term survival (1–2 years) often defined by entry criteria. This is also seen among patients in whom the decision is individualized. All patients in the cohort were being considered for lung metastasectomy and therefore had favourable features. This provides for a further bias in addition to the selection of patients on prognostic features. The median interval from registration to elective metastasectomy was 21 days with the upper quartile of delayed decisions ranging from 39 to 431 days. Lung metastasectomy is used highly selectively, not undertaken for imminently life‐threatening disease. The time needed to check that there are no further metastases or disease at other sites allows for a further manifestation of selection bias seen in the initial parts of the curves in Figure 5B,C. This effect carries over to the initial period after the metastasectomy as seen by the initial plateaus in Figure 6 after deaths on the day of operation. We suggest that this is a form of guarantee‐time bias [13]. It can occur when survival is timed from enrolment, and is compared across groups defined by a classifying event occurring sometime during the subsequent passage of time. In this case, patients who show other sites of disease, or other adverse features, fall outside the standard criteria for metastasectomy [14]. But metastasectomy remains an option for patients whose progression trajectory indicates the likelihood of longer survival. It is carried out if survival appears to be ‘guaranteed’ for a reasonable length of time. In assessment for liver metastasectomy there is a period of assessment before the final decision is made. It is a conscious policy referred to by liver surgeons as the ‘test of time’ [15, 16].

The early deaths in the elective metastasectomy stratum merit attention. There were three deaths on the day of surgery and a further five in the 6 months after operation. Although lung metastasectomy is comparatively low risk, thoracic surgery is associated with non‐trivial rates of pneumothorax, persistent air leak, bleeding, lung infection and pleural space infection, all of which take their toll on wellbeing and may contribute to deterioration and earlier death than would otherwise have been the case. Deaths in the months after operation for selected patients with very limited CRC are probably related to surgery. A similar inference was drawn in the analysis of deaths within 90 days after primary resection in the National Bowel Cancer Audit [17].

The relatively low hazard ratio of 1.19 for multiple vs. single metastases in Table 6 is not directly comparable with results derived only from metastasectomy patients. In the meta‐analysis of 24 reports including 2589 patients already referred to, the hazard ratio for multiple vs. solitary metastases, for patients having a metastasectomy, was 2.04 [9]. The better survival of patients with a solitary metastasis has been found repeatedly in case series, in the International Registry of Lung Metastases [18] and in systematic reviews [19, 20]. Figure 4 shows that among patients selected for the elective metastasectomy stratum the solitary metastasis rate was nearly double that in the unoperated patients (69% vs. 35%). A large difference was seen in survival after 2 years among patients with a difference in this risk factor.

The analysis of the randomized stratum in PulMICC produced an HR of 2.15 which is comparable with that in the meta‐analysis, while there was no evidence of an effect in the elective metastasectomy patients. A likely explanation is that in more recent practice, aware of the hazard of multiple metastases, the MDT will only recommend metastasectomy if the balance of other risk factors is favourable. In randomized patients, minimization prevents trading off risk factors.

However, erosion of the effect of a risk factor as the practice becomes more selective has been observed in the use of risk factors in case selection [21]. A simple analogy might help explain. Looking at performance data of a large pool of high school basketball players an analyst noted that youths ≥2 m tall were higher scorers. They were preferentially picked for the county team. When the analysis was run for elite teams, height was no longer such a strong discriminator. They were nearly all very tall. Blackstone and Lauer have called this effect ‘work up bias’ [22]. The coach, however, continued to select for the squad one or two players ≥1.7 m tall who could almost unerringly shoot and score from 15 to 20 m away. As does the coach, an MDT looks at all the factors. If a patient has two to three metastases that have remained much the same over a year of observation, they know that patient is likely to have a longer survival.

## DISCUSSION

The major limitation of the PulMiCC cohort study is that it is not a randomized comparison and cannot provide evidence about survival benefit attributable to metastasectomy. The best currently available data are in the PulMiCC RCT report [1].

We can confidently state that the 5‐year survival of people with lung metastases from CRC is not zero. Among 481 patients in the cohort, 169 patients did not have a metastasectomy and 37 of them were 5‐year survivors (22%, 95% CI 16%–29%). It is possible but very unlikely that a few of these survivors did not in fact have malignant lung lesions. We specifically sought information on long survivors, however treated, and the PIs did not report any case in whom the diagnosis of CRC had been wrong.

In most cases after lung metastasectomy CRC recurs sooner or later, but it is quite feasible that in some cases the lung metastases are the only residual site of disease and metastasectomy is curative. We hear anecdotal accounts [23] but documented proof of disease‐free survival at a long interval after lung metastasectomy are yet to be seen. Reported long‐term survivors have usually also had systemic treatments. The majority of patients in this cohort with and without metastasectomy had systemic treatments. An analysis of those treatments is the subject of a further report.

The big data study in the English NHS already referred to showed that lung metastasectomy is highly selective, being used on only 2.3% of patients with resected CRC in 2013 [10]. A study in the Korean National Health Insurance Database of 2573 CRC lung metastasectomy patients found a similar rate of 2.5% [24]. The lung metastasectomy operations referred to as ‘a pillar of modern thoracic surgery’ were performed at a rate of about one a month in the two series cited in support [5]—more a flying buttress than a pillar. This is a highly selective practice.

At this point, discussants of observational studies tend to point to ‘the need for trials’. In this instance we refer to the PulMiCC RCT, a trial already done. It refutes the zero assumption and substantially narrows the plausible effect size from the assumed 40% difference.

The National Institute for Health and Care Excellence (NICE) has considered the question of lung metastasectomies and the recommendation was to ‘consider’ metastasectomy. While it might have been intended as a weak recommendation, it sends out a signal for ‘business as usual’. The guideline development group advising NICE discounted PulMiCC as too small [25]. Instead, they chose to use a non‐randomized follow‐up study in which 48 had metastasectomy. PulMiCC's metastasectomy arm fell short of their unspecified cut‐off with only 46 patients but there was a control group. Also recommended for consideration was peritoneal surgery. They cited an RCT in support. It had the same surgery in both arms but the authors concluded ‘that high‐quality surgery is of value’ which cannot be derived from the abstract cited. The position with respect to liver resection was summed up by surgeons at Memorial Sloan Kettering: ‘We took as our point of departure the assumption that there will never be an RCT to answer the question of if liver resection has a role in the management of CRLM [colorectal liver metastases], or even to quantify its exact benefits’ [26] It seems questionable that the practitioners of a particular form of surgery should seek to bar the way to its evaluation. We are aware that the IMPACT initiative of ACPGBI includes active detection of metastases for resection but meta‐analysis of the many trials of more vs. less intensive screening protocols have found no overall survival benefit [27, 28].

Thoracic surgeons have addressed the question of the clinical effectiveness of lung metastasectomy by participating in the PulMiCC studies, the RCT and this cohort, but resolution of this matter is unlikely to come from specialist thoracic surgeons engaged in a very small part of the overall treatment of advanced CRC. ACPGBI have made a commitment to improving the care of patients with advanced CRC [11]. That should include reducing and avoiding the use of ineffective treatments if only to make room in the budget for adopting new ones. It is noteworthy that, in the process of prioritizing patients for cancer treatments during the COVID‐19 pandemic, metastasectomy was deemed low priority. The use of stereotactic radiotherapy to treat ‘oligometastases’ has been commissioned by NHS England on the basis of very weak evidence, and this may well supersede the use of surgical metastasectomy [29].

Colorectal MDTs are in the best position to implement trials of treatments for advanced CRC. Commenting on yet another round of the homoeopathy vs. allopathy debate *The Lancet* recognized that allopathy might have RCT evidence but homoeopathy has a following and while ‘doctors need to be bold and honest with their patients about homoeopathy's lack of benefit’ they should also be honest ‘with themselves about the failings of modern medicine to address patients' needs for personalized care’. The IMPACT initiative seeks to personalize care [11] but all systemic treatments have been introduced on the basis of controlled trial evidence. To paraphrase *The Lancet*, doctors need to be bold and honest with themselves about the failure to even seek proof of the effectiveness of local treatments. This is a declared research priority of the ACPGBI [12].

## CONFLICTS OF INTEREST

None of the authors has a conflict of interest with respect to any of the content of this submission.

## ETHICS STATEMENT

Ethics approval was granted by the National Research Ethics Committee London—Hampstead 10/H0720/5. Approval for follow‐up as an audit of practice 11 Feb 2019 HAMPSTEAD, NRESCommittee. London‐(HEALTH RESEARCH AUTHORITY).

## AUTHOR CONTRIBUTION

Trial design, leadership and management: TT, VF, FM, LF, PL. Principle investigators TB, MM, JK, YZ. Trial coordination CNG NRW. Manuscript drafting and editing TT, VF, FM. All authors have seen draft versions and the final version as submitted.

## Data Availability

Data available on request from the authors.
